# An Unusual Presentation of Median Arcuate Ligament Syndrome

**DOI:** 10.7759/cureus.9131

**Published:** 2020-07-11

**Authors:** Aditya Mehta, Anandbir S Bath, Mohammad U Ahmed, Simran Kenth, Jagadeesh K Kalavakunta

**Affiliations:** 1 Internal Medicine, Western Michigan University Homer Stryker M.D. School of Medicine, Kalamazoo, USA; 2 Cardiology, Ascension Borgess Hospital, Kalamazoo, USA

**Keywords:** median arcuate ligament syndrome, celiac artery stenosis, anginal chest pain, chest discomfort, chronic abdominal pain

## Abstract

Median arcuate ligament syndrome (MALS) is a rare benign condition typically affecting young females. It usually presents with abdominal symptoms of pain, nausea, and unintentional weight loss. They are usually diagnosed incidentally on CT of the abdomen done for abdominal pain. Here we present a rare case of MALS which presented with an anginal type of chest pain without any abdominal symptoms leading to an extensive workup and incidental diagnosis.

## Introduction

Abdominal pain is a frequent reason for visits to the emergency department (ED) and it may be a potential manifestation of a cardiac condition. The median arcuate ligament (MAL) is located at the T12-L1 level and is formed by the bridging fibers of the left and right crura of the diaphragm. These fibers border the aortic hiatus anteriorly, with the aorta, azygos vein, and thoracic duct passing through the aperture [1-3]. Median arcuate ligament syndrome (MALS) is a condition that occurs when fibers of the MAL forming the aortic hiatus compress the celiac trunk, its branches, or other neurogenic structures [4,5].

MALS is a benign condition that affects approximately two per 100,000 patients. These patients are typically women aged 20-60 years with a thin body habitus and a three-month to ten-year history of symptoms [6,7]. MALS presents with nonspecific symptoms of mesenteric ischemia: postprandial epigastric pain, nausea, vomiting, bloating, diarrhea, and unintentional weight loss due to food aversion. Epigastric abdominal bruits may also be auscultated on the physical exam. The condition can be diagnosed with computed tomography angiography (CTA), magnetic resonance angiography (MRA), or duplex ultrasonography. Treatment typically consists of surgical or endovascular techniques [6,7].

## Case presentation

Our patient is a 71-year-old woman who had been experiencing intermittent episodes of chest discomfort for multiple years. She had a past medical history of uncontrolled hypertension, dyslipidemia, hypothyroidism, obstructive sleep apnea, asthma, and tobacco use. Her past surgical history was significant for laparoscopic cholecystectomy and appendectomy. These episodes were described as a “band of chest tightening” with intermittent radiation to the jaw. The patient endorsed exertional shortness of breath, dizziness upon standing, frequent headaches, and palpitations. She also described fluctuation in her blood pressure with high systolic blood pressures ranging between 160 to 170 mmHg. A documented maximum systolic pressure of 205 mmHg was also reported. She denied orthopnea, paroxysmal nocturnal dyspnea, claudication, lower extremity edema, and syncope.

Laboratory work-up was negative for hematologic, renal, or cardiac abnormalities. Electrocardiogram demonstrated regular rate, normal sinus rhythm, and poor R-wave progression which was thought to be secondary to her obesity. The chest X-ray was unremarkable for acute changes. Echocardiography and two weeks of cardiac event monitoring were performed to assess ventricular systolic function and presence of arrhythmias, respectively. Both of these studies were negative for significant disease. Myocardial perfusion imaging and CT coronary angiography studies conducted several years prior were negative for signs of ischemia. A nuclear stress test was obtained which turned out to be unremarkable with no signs of ischemia or infarct. Bilateral renal artery ultrasound was done to evaluate renal artery stenosis as a cause of the patient’s persistent hypertension and was negative. 

Despite extensive investigations and evaluation for the cause of her chest discomfort, no underlying etiology could be identified. A decision was made to perform left and right cardiac catheterization which demonstrated only mild atherosclerotic disease (20%-30%) in the mid-portion of the left anterior descending (LAD) artery without any significant coronary artery obstruction (Video 1).

**Video 1:** The video shows the left anterior descending artery (LAD) with mild (20%-30%) atherosclerotic disease in the mid-portion with no significant stenosis. The left circumflex artery (LCx) also appears to be free of any significant disease.

**Figure 1:**
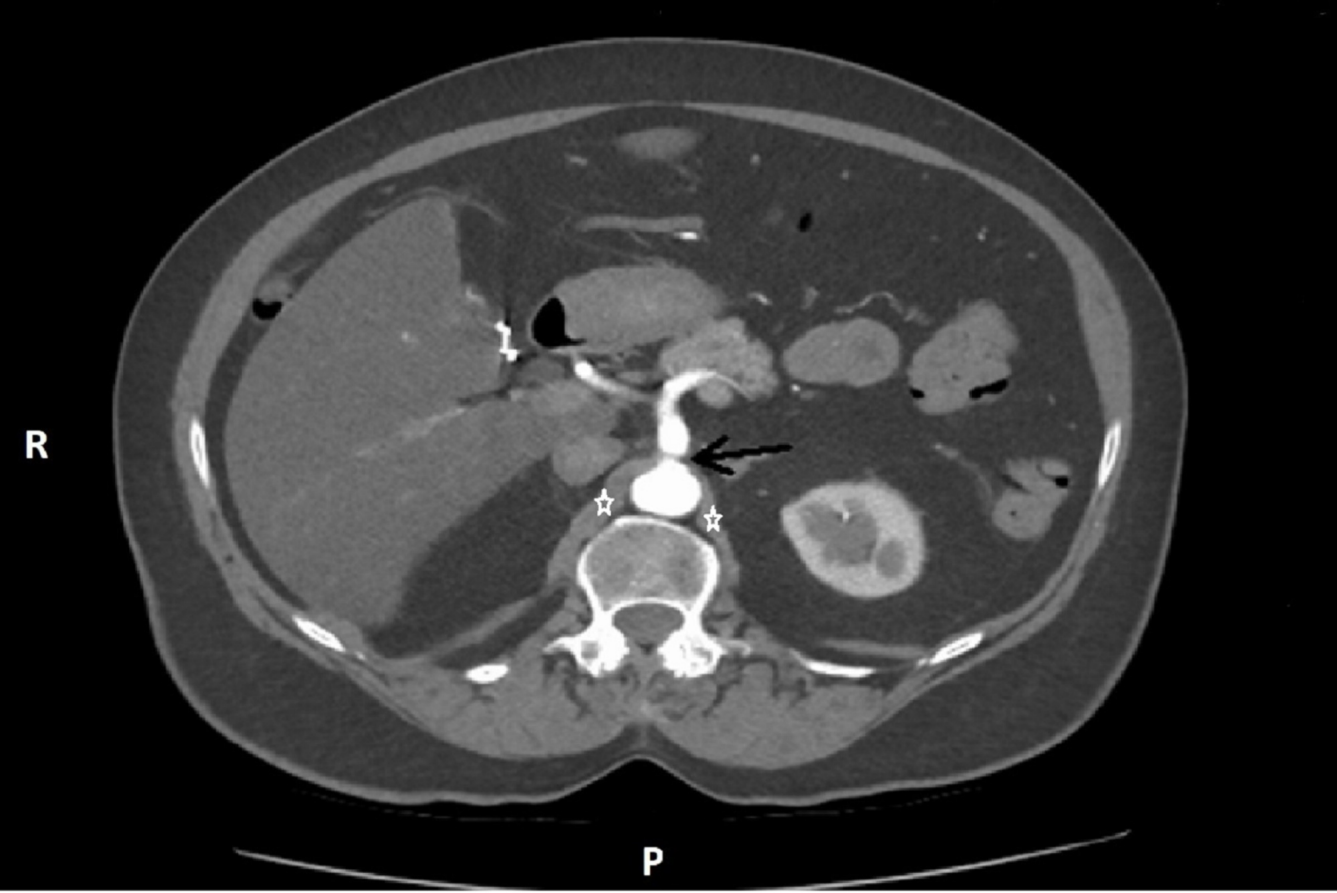
Axial CT angiogram of the abdomen. The image at the level of origin of celiac trunk (black arrow), in which a linear hypodense structure (star) separates it from the abdominal aorta and indicates the arcuate ligament compression

**Figure 2:**
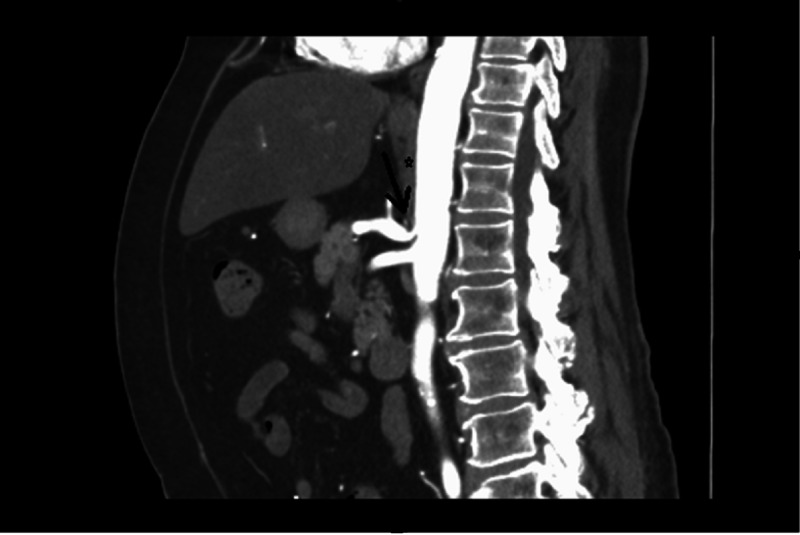
Sagittal CT angiogram of the abdomen. The image shows that median arcuate ligament (star) is located anterior to the celiac trunk and the classic compression can be seen proximal to the celiac trunk with post stenotic dilatation (black arrow).

Given the indeterminate cardiac workup, an outpatient gastrointestinal referral was recommended. The patient reported to her gastroenterologist that she had been experiencing postprandial abdominal discomfort that was unrelated to any specific diet. This abdominal discomfort radiated to her chest, neck, and back with associated nausea and occasional emesis. An upper gastroesophageal endoscopy was performed for suspicion of peptic ulcer disease which revealed only mild antral gastritis with biopsy negative for Helicobacter pylori infection. Subsequently, a CT of the abdomen with an angiogram was obtained, which demonstrated moderate-to-severe stenosis of the origin of the celiac artery with post-stenotic dilatation (Figures 1-2).

These findings led to the suspicion of MALS. The patient underwent a celiac angiogram which revealed a 30% stenosis of the celiac artery at peak inspiration and 70% stenosis at peak expiration. Her pressure gradients between peak inspiration and expiration were significant for moderate to severe celiac artery stenosis with post stenotic dilatation. 

Subsequently, the patient underwent robotic-assisted laparoscopic median arcuate ligament release surgery after which her symptoms resolved for a short period of time. Eventually, because of her persistent symptoms, she was referred for celiac artery stenting. Three months after the procedure, she was seen in the clinic for follow up. At that time, her food fear, nausea, and abdominal pain had completely resolved and she remained pain-free.

## Discussion

MALS is a rare condition that presents with abdominal discomfort, nausea, vomiting, bloating, unintentional weight loss secondary to food aversion, and epigastric abdominal bruits. It should be noted that while epigastric bruits may be auscultated in 85% of patients, this finding may also be discovered in 30% of healthy adults [6,7]. Demographic reports vary but the condition typically affects women aged 20-60 years of age with thin body habitus. The female to male ratio of MALS is reported to be 3:1 [1]. The reason for this predominance in women remains unclear but may be due to a predilection for the celiac artery to emerge more cephalad in women as compared to men [8]. Due to its benign nature, nonspecific symptomatology, and significant overlap with other conditions, patients will typically present with a history of an extensive workup to rule out other conditions. MALS is a diagnosis of exclusion, and oftentimes multiple investigations are required to rule out other pathologies such as esophagitis, cholelithiasis, pancreatitis, etc [1].

The pathophysiology of this condition remains to be elucidated. The cause is most likely multifactorial, combining the effects of celiac artery compression and subsequent foregut ischemia, with celiac plexus and/or celiac ganglion irritation and subsequent splanchnic vasoconstriction to cause the presenting symptoms [1,6,7,9,10]. This multifactorial etiology appears to be supported by a study demonstrating that correction of celiac artery compression alone results in 53% of patients being asymptomatic on long-term follow-up, whereas combined release and revascularization results in 79% of patients being asymptomatic on long-term follow-up [11].

The diagnosis of MALS may be made with multiple imaging modalities, including MRA, CTA, Doppler ultrasonography, selective angiography, and gastric exercise tomography. The celiac artery stenosis is respiratory-dependent and may require respiratory maneuvers for an accurate diagnosis. Most typically, a hook-like downward displacement of the celiac artery with post-stenotic dilatation during expiration, as shown in Figure 2, is indicative of diagnosis [12]. The treatment is typically done with an open or laparoscopic surgical release. Other options also include endovascular stenting, bypass grafting, or percutaneous transluminal angioplasty. Surgical release and vascular intervention combined typically yield the best results, and patients should be counseled on the potential need for multiple interventions to maintain an asymptomatic prognosis [6,7,9,10]. In our patient, the surgical release was not enough for symptom resolution, and the patient eventually ended up requiring endovascular stenting for persistent symptoms. The combined approach of treatment ultimately relieved the patient of her symptoms.

Our patient was an overweight woman at the age of 71, which is just outside the typical demographic of younger women with thin body habitus. Although she had been experiencing symptoms for several years, this specific case is unique in that her initial presenting symptoms seemed to be chiefly cardiac in origin. Her chest pain radiating to her jaw, palpitations, and exertional shortness of breath prompted a detailed cardiac workup that eventually turned up indeterminate and required a gastroenterologist referral. Despite being overweight, suspicion of celiac artery atherosclerosis is a consideration but given her CT angiogram findings, it did not reveal any atherosclerosis in the celiac artery.

This case provides an excellent example of the complicated picture that MALS can present with, and the extensive work-up it requires before making this diagnosis of exclusion. It is important to keep this diagnosis in mind when patients present in a similar fashion, but its benign nature requires more acute and serious conditions to be ruled out first.

## Conclusions

MALS is a rare, benign condition that causes recurrent abdominal symptoms. It is a diagnosis of exclusion and requires a thorough work-up to rule out other acute, more serious etiologies. Due to its nonspecific symptomatology, it can masquerade as other pathologies including, although rarely, those of cardiac etiologies. After meticulous exclusion of other etiologies, surgical referral is necessary for treatment. It often requires a multidisciplinary approach with a team involving subspecialists comprising of a gastroenterologist, vascular surgeon, and general surgeon.
